# Identification of microRNAs in Wool Follicles during Anagen, Catagen, and Telogen Phases in Tibetan Sheep

**DOI:** 10.1371/journal.pone.0077801

**Published:** 2013-10-17

**Authors:** Guangbin Liu, Ruize Liu, Qinqun Li, Xiaohui Tang, Mei Yu, Xinyun Li, Jianhua Cao, Shuhong Zhao

**Affiliations:** 1 Key Laboratory of Agricultural Animal Genetics, Breeding and Reproduction, Ministry of Education, Huazhong Agricultural University, Wuhan, Hubei, China; 2 Agriculture and Animal Husbandry College of Tibet, Linzhi, Tibet, China; University of California, Davis, United States of America

## Abstract

**Background:**

Wool quality is one of the most important economic traits in sheep. The wool fiber is derived from specialized skin cells that are referred to as wool follicles. To understand the roles of microRNAs (miRNAs) in wool fiber growth, we detected the expression patterns of miRNAs in wool follicles at the anagen, catagen, and telogen stages from Tibetan sheep through Solexa sequencing.

**Results:**

A total of 244 mature miRNAs were identified. Of these, only five miRNAs are listed in the database of sheep miRNAs (miRBase Database V19), and the other 239 miRNAs have not been previously described in this species. Further analyses indicated that 204 miRNAs are evolutionarily conserved among mammal species, whereas 35 of the identified miRNAs were first found specifically in sheep. The expression pattern analyses showed that the expression levels of 39, 34, and 20 of the miRNAs significantly change between anagen and catagen, between anagen and telogen, and between catagen and telogen, respectively. The results of the bioinformatics analysis show that these differentially expressed miRNAs might regulate wool follicle development by targeting genes in many different pathways, such as the MAPK and Wnt pathways, as well as the pathways that regulate the actin cytoskeleton, focal adhesion, and tight junctions. Furthermore, we identified six differentially expressed miRNAs (oar-miR-103-3P, oar-miR-148b-3P, oar-miR-320-3P, oar-miR-31-5P, oar-novel-1-5P, and oar-novel-2-3P) that might target the key genes of the Wnt pathway. It has been reported that the Wnt pathway is critical for wool follicle development. Therefore, these miRNAs may regulate wool development through the Wnt pathway.

**Conclusions:**

Our results provide new information on the identification and expression pattern of miRNAs in wool follicles. Our data might therefore aid in the understanding of the mechanisms of wool follicle development in sheep.

## Introduction

MicroRNAs (miRNAs) are a class of noncoding small RNAs. A mature miRNA is usually single-stranded and 21-24 nt (nucleotides) in length. It can bind the 3’UTR of mRNA through pairing with the miRNA seed region and can block gene expression by inhibiting the translation or degradation of the mRNA [1]. Researchers have revealed that a miRNA can target many different sites on the same or different genes and that approximately 30% of genes are regulated by miRNAs [2]. Since the first miRNA was discovered in 1993 [3], thousands of miRNAs have been identified in different species. Increasing evidence shows that miRNAs participate in many biological processes, particularly cell proliferation, differentiation, apoptosis, and immune responses [4].

Wool, as one of the most valuable products from sheep, is an important material in the textile industry. An improvement in the wool quality will result in marked economic value in the field of animal husbandry. As the direct tissue from which wool is derived from, the wool follicle plays a vital role in the production of better-quality wool [5]. In general, the development of the wool follicle could be divided into three stages: anagen, catagen, and telogen. During these three stages, the wool follicle undergoes growth, regression, and rest phases [6]. The hair follicle is also a regenerating system, and each mature hair follicle develops under a growth cycle [7-10].

Some recent reports have suggested that miRNAs might be involved in hair follicle development. For example, miR-31 has been proven to play important roles in hair matrix differentiation and hair shaft formation [11]. In addition, studies have indicated that miRNAs could be important regulatory factors in hair follicle development. However, the molecular mechanism of miRNAs in hair follicle development has not been illustrated. 

To understand the functions of miRNAs in wool follicle development, wool follicles at the anagen, catagen, and telogen stages were collected in this study. The miRNAs of wool follicles and the expression patterns of these miRNAs during the anagen, catagen, and telogen stages were investigated through Solexa sequencing. A number of miRNAs were found to be differentially expressed between the three hair follicle developmental stages. Our study could provide new knowledge regarding the development of wool follicles in the sheep. 

## Results

### Overview of the Solexa sequencing data

**Figure 1:**
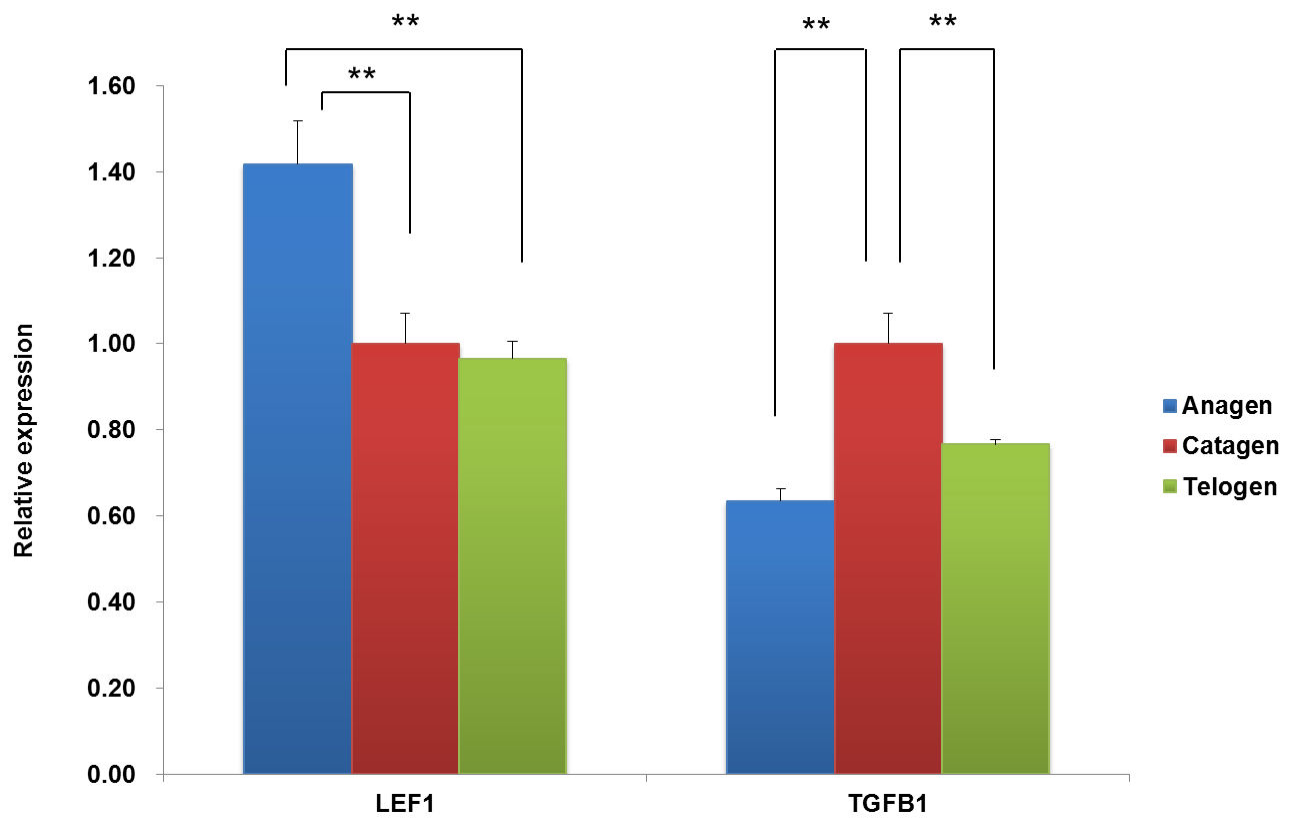
Confirmation of the samples from the three phases through detection of the expression of marker genes. The two maker genes LEF1 and TGFB1 were detected by QPCR. The LEF1 gene was more highly expressed in anagen than in catagen and telogen (P < 0.01), and the TGFB1 gene was more highly expressed in catagen than in anagen and telogen (P < 0.01).

**Figure 2:**
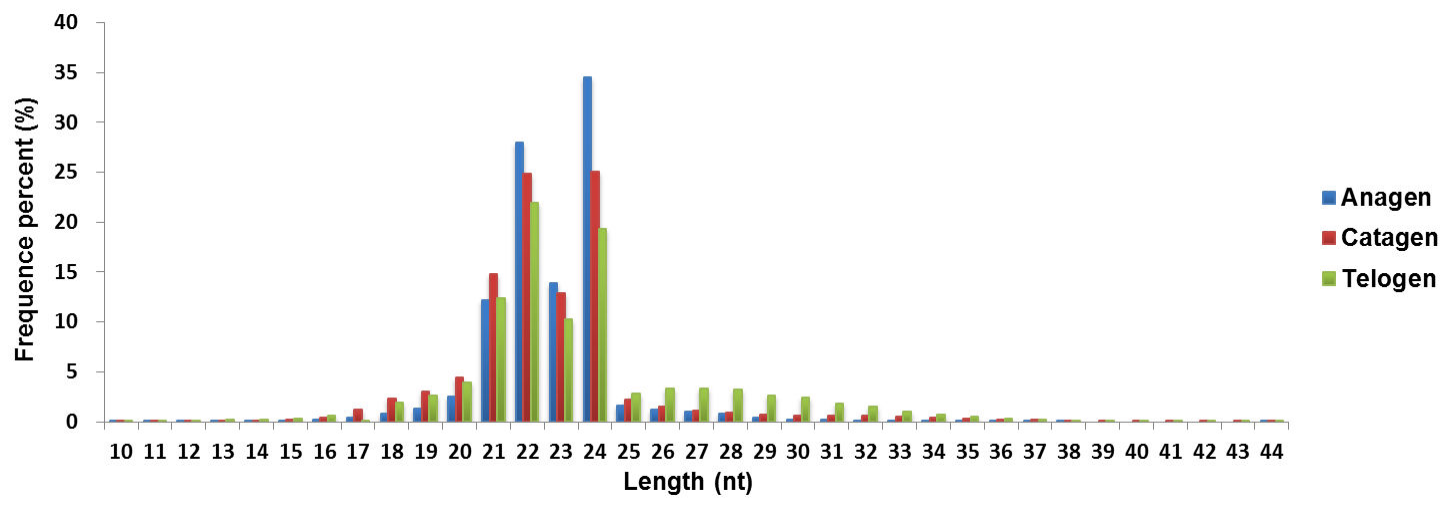
The distribution of the read length from the Solexa sequencing data. The majority of the reads in the three libraries are 21-24 nt in length.

To understand the expression pattern of miRNAs during wool follicle development, three small RNA libraries were constructed from the total RNA of wool follicles at the anagen, catagen, and telogen stages. Each library pooled the RNA of the wool follicles at the same phase from three Tibetan sheep. We detected the expressions of two marker genes, LEF1 and TGFB1, to confirm that our samples were collected from the right phases. Previous studies have reported that the expression of LEF1 in hair follicles is higher in anagen compared with the catagen and telogen phases, whereas the expression level of TGFB1 is upregulated in catagen and downregulated in telogen [12,13]. The QPCR (Real-time Quantitative PCR) results (Figure **1**) show that the expression patterns of these two genes are consistent with the expected phases. Subsequently, Solexa sequencings were performed for each library. A total of 12,740,200 (anagen), 16,768,947 (catagen), and 16,564,009 (telogen) raw reads were obtained from the three small RNA libraries. After removing the low-quality reads and adapter fragments, 12,197,976 (anagen), 15,623,402 (catagen), and 15,079,495 (telogen) clean reads were obtained, which corresponded to 97.37%, 95.05%, and 92.81% of the raw reads from the three small RNA libraries, respectively. The length distribution of these clean reads is shown in Figure **2**. The majority of the reads are 21-24 nt in length, and the number of 22-nt and 24-nt reads are significantly greater than the number of reads of other sizes. This result coincides with the length range for Dicer-derived products.

### Identification of miRNAs from sequencing data using the miRDeep software and annotation

The sequencing data from the three small RNA libraries were analyzed using the miRDeep software (Version 2.0.0.3) (http://mdc.helmholtz.de/8551903/en/research/research_teams/systems_biology_of_gene_regulatory_elements/projects/miRDeep). The reference genome sequence of sheep used in this study was OAR2.0 Released by CSIRO Australia (http://www.livestockgenomics.csiro.au/), and the miRNA database used is the miRBase database V19 (http://www.mirbase.org/). In total, 244 mature miRNAs were identified, and these miRNAs belong to 208 unique miRNA precursors. In addition, 218, 229, and 221 miRNAs are expressed in the anagen, catagen, and telogen phases, respectively. Of these, 198 miRNAs overlapped among the three libraries, whereas seven, eight, and three miRNAs are specifically expressed in anagen, catagen, and telogen libraries, respectively. 

**Figure 3:**
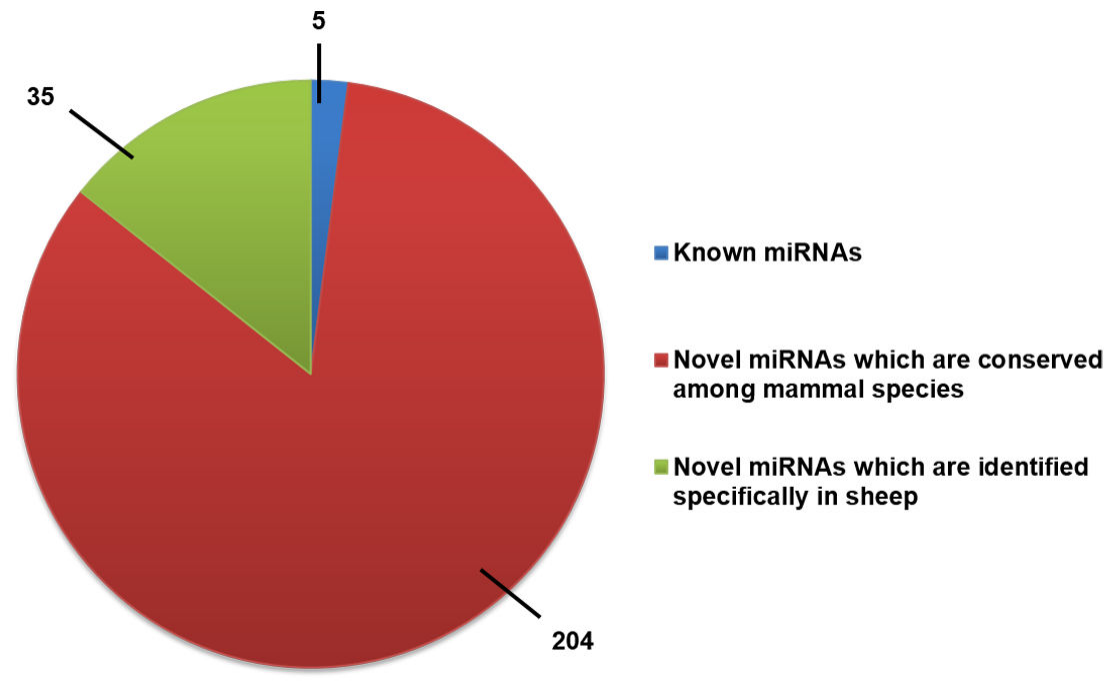
Annotation of miRNAs. The number of miRNAs is indicated.

The annotation work was performed using the miRBase database V19 (Figure **3**). Only five of the sheep miRNAs in our dataset have been deposited into the miRBase database V19: oar-miR-379, oar-miR-127, oar-miR-411a, oar-miR-493, and oar-miR-382. The other 239 miRNAs have not been reported in sheep (miRBase database V19). Among these 239 candidate miRNAs, 204 exhibit evolutionary conservation with the mature miRNAs from other species, and 35 miRNAs are novel candidates. All of the information on the 244 mature miRNAs is shown in Table **S1**, and we list the 10 most abundant miRNAs in Table **1**. We found that the expression levels of these 10 miRNAs constitute approximately 90% of the total expression level of all of the miRNAs. Oar-miR-146a-5P is the most abundantly expressed miRNAs in wool follicles. Its expression level is nearly six-fold higher than that of the second most abundantly expressed miRNA (oar-let-7a-5P) in our data, and constitutes more than a half of the total expression level of all of the miRNAs.

**Table 1:** The 10 most abundantly expressed miRNAs in sheep wool follicles.

	Expression level			
miRNA name	**Anagen**	**Catagen**	**Telogen**	**Mature sequence**
oar-miR-146a-5P	350813.36	231387.31	180345.50	ugagaacugaauuccauagguugu
oar-let-7a-5P	66038.41	32012.43	29733.22	ugagguaguagguuguauaguu
oar-miR-24-3P	30573.84	21231.16	19627.45	uggcucaguucagcaggaacagg
oar-let-7f-5P	41098.79	13744.00	15506.35	ugagguaguagauuguauaguu
oar-miR-378-3P	18057.42	17657.68	15197.59	acuggacuuggagucagaaggc
oar-miR-21-5P	16367.80	14890.42	11769.62	uagcuuaucagacugauguugac
oar-miR-27b-3P	10270.23	7511.62	6515.14	uucacaguggcuaaguucugc
oar-let-7c-5P	6591.42	4297.40	4593.59	ugagguaguagguuguaugguu
oar-miR-184-3P	4098.79	4627.74	5216.75	uggacggagaacugauaagggu
oar-miR-103-3P	6985.91	3553.20	2891.08	agcagcauuguacagggcuauga

**Figure 4:**
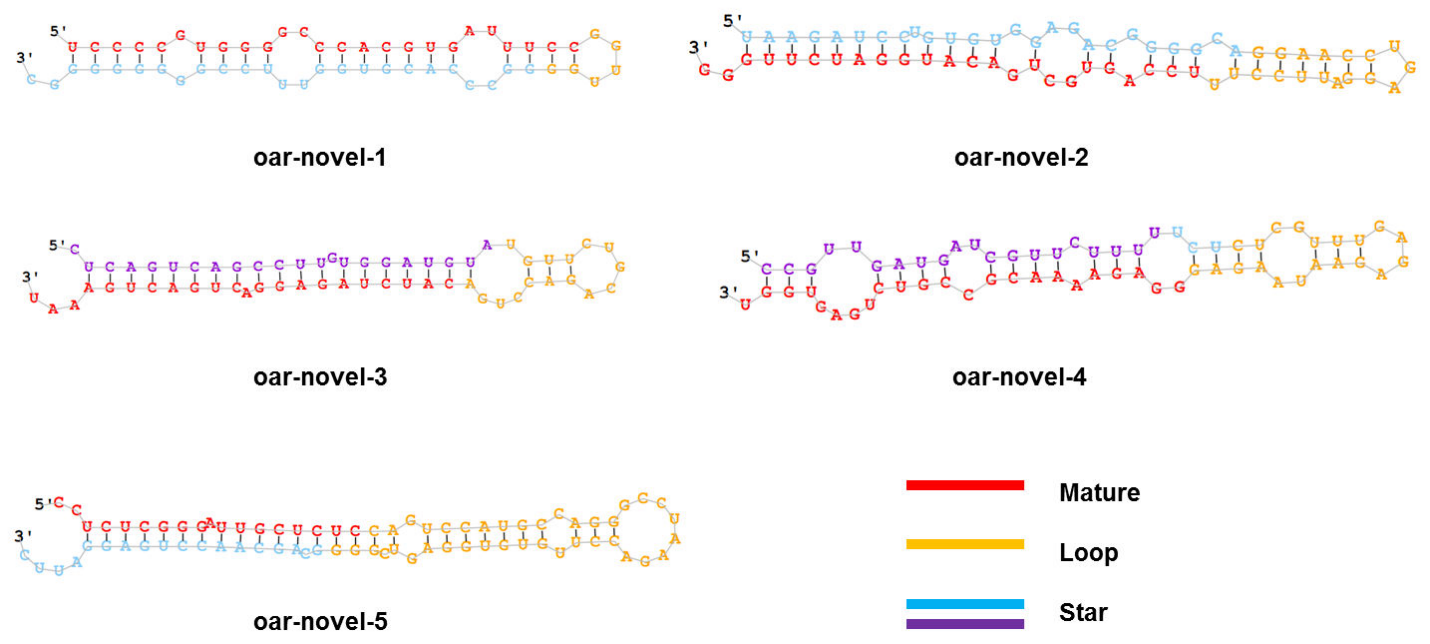
Predicted secondary structures of novel miRNAs. The red color indicates the mature sequence, the yellow color indicates the loop sequence, the blue color indicates the predicted star sequence, and the purple indicates the miRNA star sequences.

The precursor sequences and secondary structures of the 35 novel miRNAs identified from our sequencing data using miRDeep software (Table **S2**) were predicted (Figure **S1**). The five most abundantly expressed novel miRNAs are shown in Tables **2** and **3** and Figure **4**. The results show that most of the novel miRNAs in our dataset exhibit relatively low expression levels. In addition, many of the novel miRNAs present a phase-specific expression pattern. For example, oar-novel-1 is specifically expressed in catagen, whereas oar-novel-2 is not detected in anagen. In fact, 23 of the novel miRNAs are either expressed or not expressed in a phase-specific manner. This number accounts for 65.7% of the total number of novel miRNAs in our dataset.

**Table 2:** The five most abundantly expressed novel miRNAs in sheep wool follicles.

	Expression level				
miRNA name	**Anagen**	**Catagen**	**Telogen**	**Mature sequence**	**Rank**
oar-novel-1-5P	0.00	543.61	0.00	uccccguggggcccacgugauuucc	52
oar-novel-2-3P	0.00	38.21	414.54	uccagugcugacauggaucuuggg	55
oar-novel-3-3P	117.07	98.76	92.05	caucuagaggacugacugaaau	68
oar-novel-4-3P	65.01	43.14	34.22	ggagaaaacgccgucugaguggu	91
oar-novel-5-5P	2.05	5.89	79.38	ccucucgggauugcucuc	101

**Table 3:** Predicted precursor sequences and genome locations of novel miRNAs.

miRNA name	miRNA precursor sequence	Chromosome	Strand
oar-novel-1	uccccguggggcccacgugauuuccgguuggggcccacgugguuuccggggggggc	OAR17	+
oar-novel-2	uaagauccuguguggagacggggcaggaaccugaggauuccuuuccagugcugacauggaucuuggg	OAR3	+
oar-novel-3	cucagucagccuuguggauguauguucugcagaccugacaucuagaggacugacugaaau	OARX	+
oar-novel-4	cguugaugaucguucuuuuucucucguuugagagaauaagagggagaaaacgccgucugaguggu	OAR11	-
oar-novel-5	ccucucgggauugcucuccaguccaugccagggccuaagaccuuguguggagucggggcagcaaccugaggauuc	OAR9	+

### Validation of the sequencing data by QPCR

**Figure 5:**
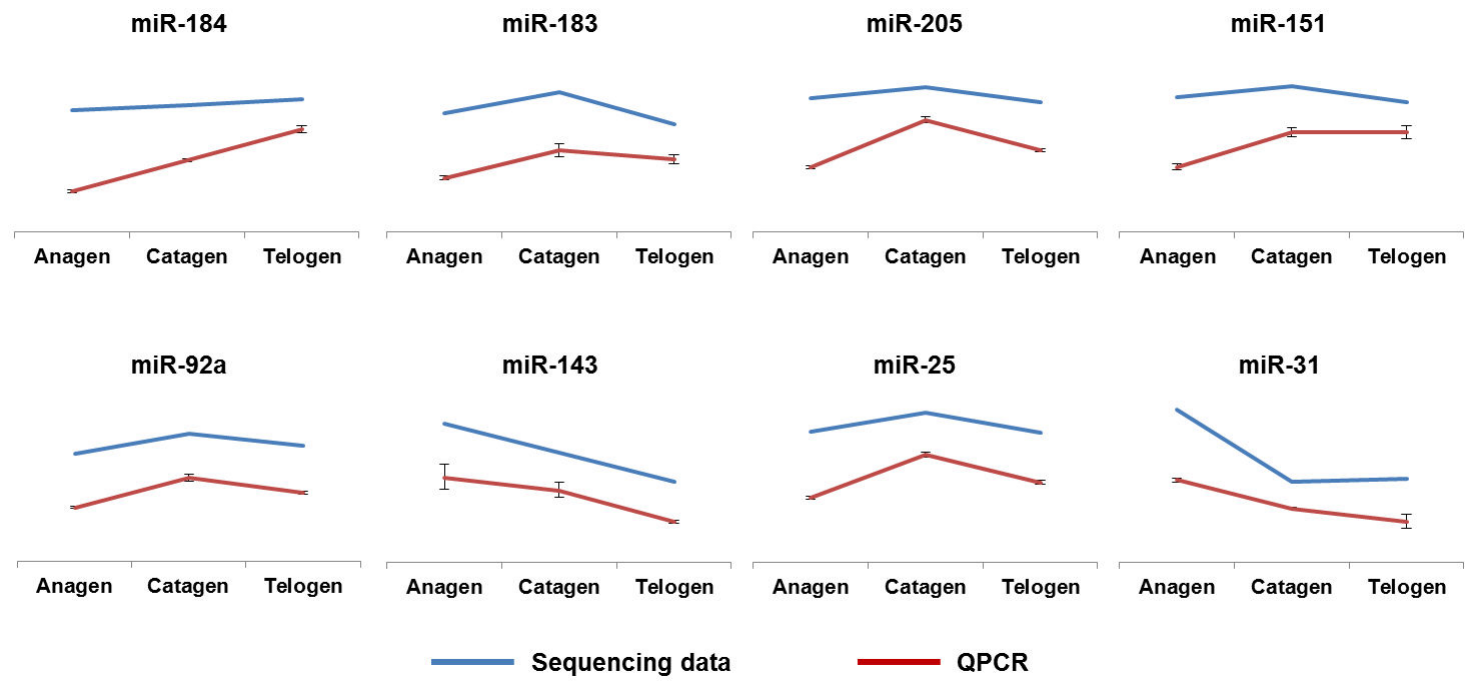
Validation of sequencing data by QPCR. The blue lines indicate the expression patterns of miRNAs in the sequencing data, and the red lines indicate the QPCR results.

 To validate the sequencing data, we randomly selected eight different miRNAs with expression levels of more than 500 per million for QPCR analyses (Figure **5**). As anticipated, the QPCR results are consistent with the sequencing data. For example, both the sequencing data and the QPCR results show that the expression level of oar-miR-184-3P is upregulated gradually from anagen to telogen. In addition, the expression level of oar-miR-183-5P is upregulated from anagen to catagen and downregulated from catagen to telogen. 

### Identification of differentially expressed miRNAs between different wool follicle development phases and prediction of miRNA target genes and pathways

**Figure 6:**
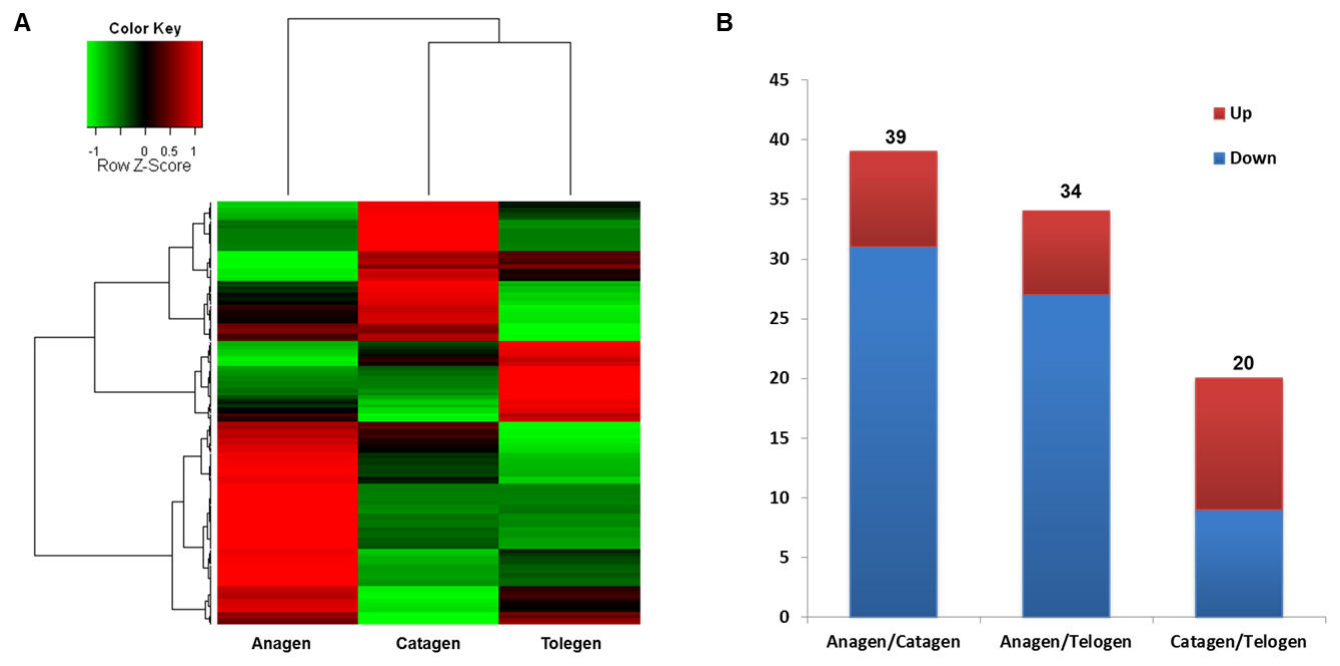
Identification of differentially expressed miRNAs among different wool follicle development phases. A: Cluster analysis of the expression levels of miRNAs in the three phases. B: The number of miRNAs that exhibited a significant change in expression level between different phases. The red color indicates upregulation, and the blue color indicates downregulation.

To understand the expression patterns of miRNAs during wool follicle development, we performed a cluster analysis (Figure **6A**). The results demonstrate that the expression levels of most of the miRNAs change during the development of the wool follicle from anagen to telogen. Approximately 50% of the miRNAs are downregulated from anagen to catagen and telogen, although a few miRNAs are upregulated in telogen. 

We then compared the expression level of each miRNA in the three phases to identify miRNAs that are differentially expressed between two different phases (anagen compared with catagen, anagen compared with telogen, and catagen compared with telogen). The miRNAs that satisfied the criteria log2FoldChange ≥ 1 or ≤ -1 and P ≤ 0.01 were denoted as differentially expressed miRNAs. As a result, we found that the expression level of 39, 34, and 20 miRNAs significantly change between anagen and catagen, between anagen and telogen, and between catagen and telogen, respectively (Figure **6B**). We list all of the differentially expressed miRNAs in Table **S3**. The results show that the number of differentially expressed miRNAs between anagen and catagen are similar to that between anagen and telogen, but higher than the number of miRNAs that are differentially expressed between catagen and telogen. Additionally, more miRNAs are downregulated as the wool follicles develop and transition from the anagen to the telogen phase. 

**Figure 7:**
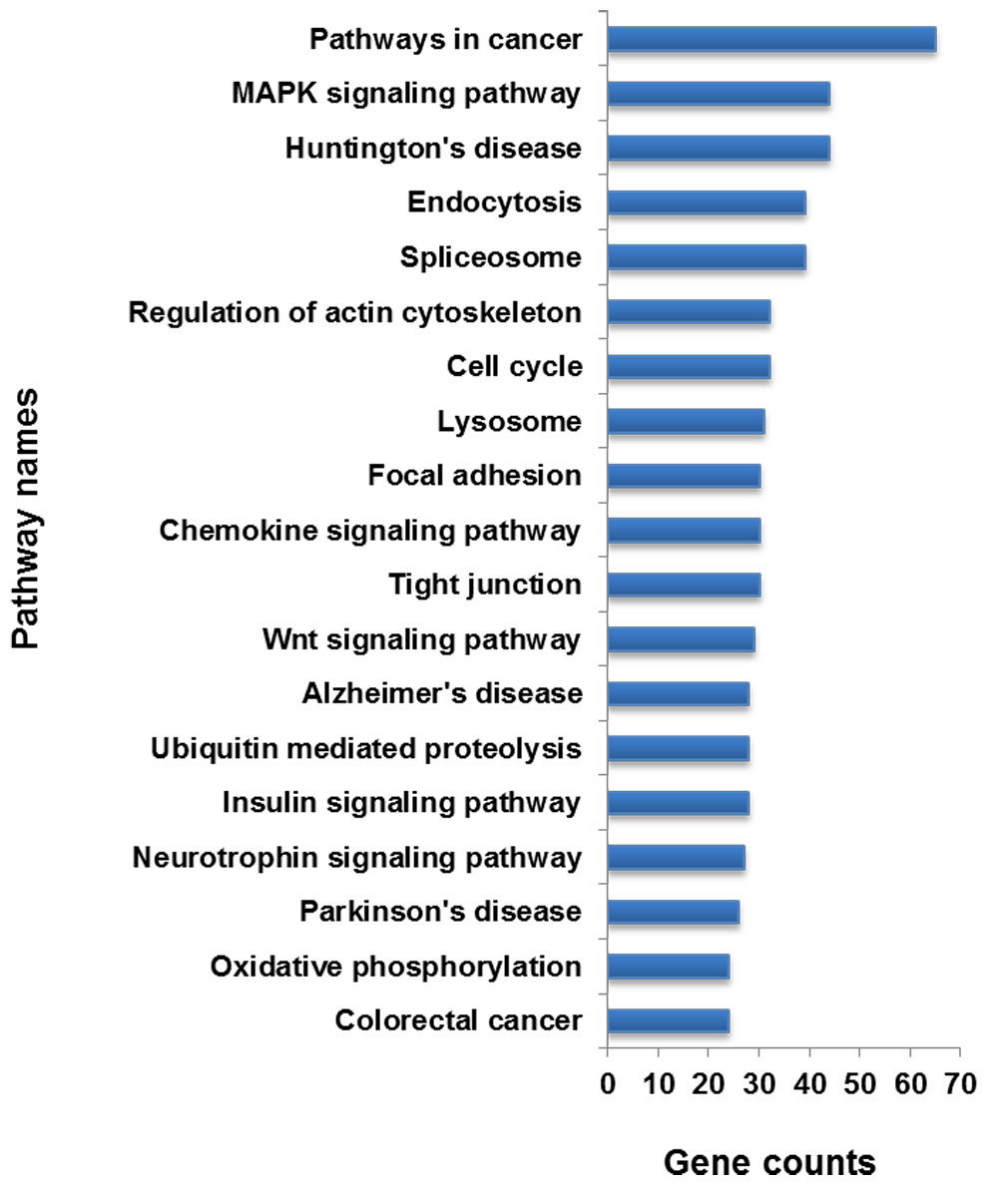
Top 20 pathways predicted to be targeted by differentially expressed miRNAs. The column indicates the unique gene number.

**Figure 8:**
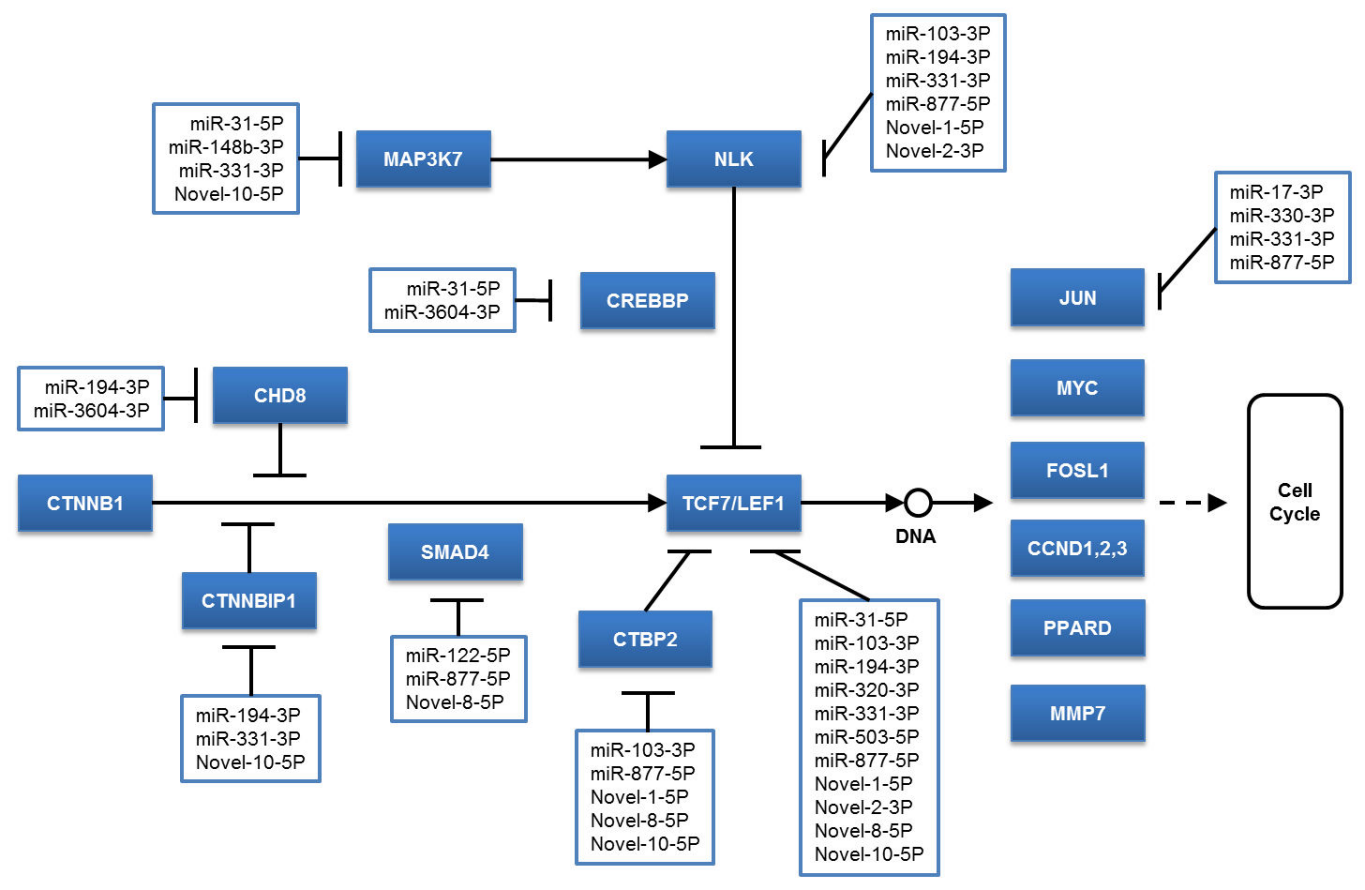
Differentially expressed miRNAs that potentially target genes in the Wnt pathway.

 To understand the function of these differentially expressed miRNAs in wool follicle development, we predicted the target genes of the miRNAs using the RNAhybrid software. A dataset of the sequenced sheep wool follicle transcriptome from a previous work (unpublished) was used as the candidate target gene reference. As a result, 3,223 genes were found to be targeted by 48 of the differentially expressed miRNAs (Table **S4**). We subsequently analyzed these 3,223 genes through the DAVID website (http://david.abcc.ncifcrf.gov/), and the results show that 176 pathways could be involved in the corresponding miRNA regulation (Table **S5**). We list 20 pathways associated with the most number of target genes in Figure **7**. Some pathways, such as the MAPK and Wnt signaling pathways, regulate cell proliferation and differentiation. We also identified pathways that are involved in the maintenance of cell and tissue structures, e.g., the regulation of the actin cytoskeleton pathway, the focal adhesion pathway, and the tight junction pathway. Of these, we focused on the Wnt pathway because many reports have revealed that the Wnt pathway can regulate hair follicle development. In our results, we found that 22 differentially expressed miRNAs can target 29 genes in the Wnt pathway (Table **4**); these miRNAs include oar-mir-16a-5P, oar-mir-31-5P, and oar-mir-103-3P. In addition, some novel miRNAs, such as oar-novel-1-5P, oar-novel-2-3P, oar-novel-5-5P, oar-novel-8-5P and oar-novel-10-5P, might be involved in the Wnt pathway. We also found the some miRNAs, such as oar-miR-103-3P, oar-miR-148b-3P, oar-miR-320-3P, oar-miR-31-5P, oar-novel-1-5P, and oar-novel-2-3P, can directly or indirectly affect the expression of the TCF7 or LEF1 genes to regulate the cell cycle (Figure **8**). 

**Table 4:** Target genes of differentially expressed miRNAs in the Wnt pathway.

	Expression level			
miRNA name	**Anagen**	**Catagen**	**Telogen**	**Target genes in Wnt pathway**
oar-miR-103-3P	6985.91	3553.2	2891.08	TCF7, NLK, MAPK8, CTBP2, AXIN1, BTRC, RHOA
oar-miR-16a-5P	4407.53	2168.61	1896.28	AXIN1, NFATC3
oar-miR-148b-3P	1483.61	470.06	623.1	AXIN1, MAP3K7
oar-miR-320-3P	1010.17	195.54	192.12	TP53, RAC1, LRP5, LEF1
oar-miR-31-5P	605.43	217.05	231.84	CREBBP, TCF7, MAPK9, AXIN1, CACYBP, MAP3K7, DAAM1
oar-novel-1-5P	0	543.61	0	TP53, TCF7, NLK, FZD1, NFATC1, PPP2R5B, CTBP2, FZD7, AXIN1, DAAM1
oar-novel-2-3P	0	38.21	414.54	TP53, NLK, LEF1, AXIN1
oar-miR-6529-5P	207	52.23	45.36	TP53, FZD1, NFATC1
oar-miR-125b-3P	62.8	5.76	65.32	TP53, SENP2, FZD1, AXIN1
oar-novel-5-5P	2.05	5.89	79.38	FZD1, PPP2R5B
oar-miR-503-5P	46.73	15.04	13.4	SENP2, TCF7, MAPK8, AXIN1, BTRC, NFATC3
oar-miR-17-3P	0.82	22.53	38.86	FZD1, JUN, NFATC3, DAAM1
oar-novel-8-5P	11.23	17.6	26.86	SMAD4, TCF7, FZD1, NFATC1, CTBP2, AXIN1, NFATC3
oar-miR-193b-3P	26.81	11.78	1.33	TP53, BTRC, DAAM1
oar-miR-122-5P	10.17	0.7	20.43	SMAD4, DAAM1
oar-novel-10-5P	0	31.24	0	DVL3, SENP2, TCF7, FZD1, LEF1, CTBP2, NFATC3, PPP2CA, CTNNBIP1, MAP3K7, DAAM1
oar-miR-330-3P	20.58	4.8	3.78	RAC1, JUN
oar-miR-331-5P	15.25	0	11.27	TP53, SENP2, FZD1, PPP2R5B, AXIN1
oar-miR-877-5P	10.82	4.03	8.36	SMAD4, MAPK8, FZD1, LEF1, CAMK2G, CTBP2, AXIN1, DAAM1, TP53, DVL3, SENP2, TCF7, NLK, PPP2R5B, JUN, BTRC, PPP2CA, NFATC3
oar-miR-3604-3P	13.53	6.27	2.72	CREBBP, CHD8, AXIN1
oar-miR-194-3P	6.15	10.5	0.66	TP53, SENP2, NLK, CHD8, LEF1, AXIN1, CACYBP, BTRC, PPP2CA, CTNNBIP1
oar-miR-331-3P	7.62	0	8.22	TCF7, NLK, MAPK8, FZD1, PPP2R5B, CAMK2G, AXIN1, JUN, CTNNBIP1, MAP3K7

## Discussion

Many studies have proven that miRNAs play important roles in cell proliferation and differentiation [4,14,15]. There is growing evidence that miRNAs take part in the regulation of hair follicle development [16]. To understand the roles of miRNAs in wool follicle development, we identified 244 mature miRNAs in wool follicles from three different hair follicle growth phases (anagen, catagen, and telogen). Compared with other species, few studies have focused on sheep miRNAs. The most recent miRBase database version 19 contains 2,042 mature miRNAs from human, 1,281 miRNAs from mouse, 755 miRNAs from cow, and only 103 miRNAs from sheep. Therefore, our study not only demonstrates the expression pattern of miRNAs during wool follicle development but also enriches the information in the miRNA database.

Our study found that miR-146a is the most abundantly expressed miRNA (in all three phases). This miRNA occupies more than 50% of the total reads (the sum of the three phases), and its expression level is nearly six-fold higher than that of oar-let-7a, which is the second most abundantly expressed miRNA in our dataset. Studies have shown that miR-146a can regulate the resolution of T cell responses [17,18] and that this miRNA also participates in inflammatory responses [19]. In addition, some studies have found that miR-146a might induce cell proliferation and differentiation [20,21]. These evidences suggested that the function of oar-miR-146a might be related to the immune response or cell growth. We predicted the target genes of miR-146a in the present study. As a result, we found that miR-146 may regulate the Notch signaling pathway by inhibiting the target gene NOTCH2. Many studies have reported that the Notch signaling pathway plays an important role in regulating the differentiation of the epidermis and hair follicles, particularly the control of cell fate in hair follicles. Members of the Notch pathway are expressed in the epidermis and hair follicles during embryonic development and the adult stage. The loss of Notch function in fair follicles leads to alopecia [22,23]. Because of the high expression level of miR-146a in all three phases, we suspect that miR-146a may take participate in the basal regulation of wool follicle development and maintenance.

In this study, we found that the expression levels of some miRNAs significantly change during wool follicle development from anagen to telogen. Further analyses show that the target genes of these miRNAs may be involved in pathways that regulate cell proliferation and differentiation and in pathways that maintain tissue and/or cell structure. These findings suggest that these miRNAs may take part in the regulation of wool follicle formation and development. Many reports have revealed that the Wnt pathway is one of the important pathways associated with wool follicle development [24]. It has been proven that the Wnt pathway plays multiple roles in cell migration, proliferation, and differentiation. When Wnt pathway was inhibited, the structure of hair follicle cannot maintain [25-28]. The LEF1 and β-catenin genes are two key factors in the Wnt pathway that could affect the cell cycle. The absence of LEF1 and β-catenin could lead to the hairless phenotype as a result of abnormal follicle formation [25,29,30]. Conversely, the overexpression of these genes in the ectoderm could induce hair follicle morphogenesis [31]. In the present study, we found 22 differentially expressed miRNAs that could be involved in the regulation of the Wnt pathway. In addition, 16 of these miRNAs may directly or indirectly regulate the expression of the LEF1 gene to control the wool follicle growth cycle. Based on the miRNA expression levels, we hypothesize that oar-miR-103-3P, oar-miR-148b-3P, oar-miR-320-3P, oar-miR-31-5P, oar-novel-1-5P, and oar-novel-2-3P, which exhibited normalized expression levels greater than 100 per million (sum of the three phases), play particular roles in the regulation of the wool follicle growth cycle. Some studies have revealed that these miRNAs participate in cell proliferation and differentiation and in cell cycle control. For example, it has been reported that miR-103 is associated with erythroid differentiation control [32] and participates in cellular migration through the regulation of CDK5R1 expression [33]. MiR-148b is related to osteogenesis [34]. In addition, miR-148a, which shares the same seed sequence with miR-148b, has been proven to promote cell proliferation by targeting p27 in gastric cancer cells [35]. These studies have indicated the miR-148b may have the same function. Some studies have shown that miR-320 is involved in cardiac injury and protection [36] and affects the cell cycles of primary murine bronchial epithelial cells exposed to benzo[a]pyrene [37]. It has been revealed that miR-31 plays an important role in the control of anagen-associated gene expression programs in the hair follicle. The absence of miR-31 results in the acceleration of the anagen phase and alterations in the hair shaft formation and outer root sheath morphology [11]. Our study revealed that oar-miR-103-3P, oar-miR-148b-3P, oar-miR-320-3P, and oar-miR-31-5P may directly or indirectly target the TCF7 and LEF1 genes, which are important transcription factors that regulate the cell cycle and could regulate the lineage differentiation of multipotent stem cells in skin [26]. Thus, we hypothesize that these four miRNAs may play multiple roles in the regulation of the wool follicle cycle. Because oar-novel-1-5P and oar-novel-2-3P are novel miRNAs found in the present study, their functions have not been previously studied. However, our study found that these two novel miRNAs might be able to regulate the cell cycle by targeting LEF1 and NLK, which is an inhibitor of LEF1. In addition, oar-novel-1-5P is specifically expressed in catagen, and oar-novel-2-3P is more highly expressed in telogen than in catagen and is not expressed in anagen. We thus hypothesize that these two novel miRNAs may take part in the regulation of phase changes in wool follicle development.

## Conclusions

We identified 244 mature miRNAs from wool follicles. Of these miRNAs, 204 are conserved with other species, and 35 are novel. In addition, we found that the expression levels of some of the miRNAs significantly change during wool follicle development, and we hypothesize that oar-miR-103-3P, oar-miR-148b-3P, oar-miR-320-3P, oar-miR-31-5P, oar-novel-1-5P, and oar-novel-2-3P may be involved in the regulation of the follicle growth cycle through the Wnt pathway. Our study could expand the database on sheep miRNAs and provide reference information on sheep and wool follicle development. 

## Materials and Methods

### Ethics statement

All research involving animals was conducted according to Regulation No. 5 of the Standing Committee of Hubei People's Congress and was approved by the Standing Committee of Hubei People's Congress and the ethics committee of Huazhong Agricultural University, P. R. China. The ethics committee of Huazhong Agricultural University, P. R. China approved this study, and the approved permit number for this study is "HBAC20091138”.

### Animal and sample collection

Three female 2-year-old Tibetan sheep from the Agriculture and Animal Husbandry College of Tibet in China were used in this study. The wool follicle bulb samples from the anagen phase were collected in May. The samples from the catagen phase were collected in November, and the samples from the telogen phase were collected in January of the following year. The wool follicle bulb samples were mixed with TRIzol (Invitrogen) immediately after collection. The samples were centrifuged at 12,000 rpm and 4°C for 10 minutes. The cleared supernatant was transferred to a fresh tube and stored at -80°C.

### Preparation of small RNA libraries and sequencing

The total RNA was isolated according to the instructions provided by the manufacturer of the TRIzol Reagent (Invitrogen). The RNA samples from the three different sheep and the same developmental phases were pooled together based on an equal RNA quantity. In the end, three RNA libraries from nine total RNA samples were built, and these represented the samples from the three different phases. Subsequently, the small RNA sequencing libraries were prepared using a “Small RNA Sample Prep Kit (Illumina)”, and all of the procedures and standards were performed according to the kit protocol. The Solexa sequencing work was performed by Beijing Genomics Institute (BGI).

### Sequencing data analysis by miRdeep2 software

The miRNA sequencing data were analyzed using the miRDeep software (version 2.0.0.3) [38]. The miRNA reference was obtained from the miRBase database (version 19; release August 2012) [39-43]. The genome reference used was the OAR V2.0 release by CSIRO Australia (http://www.livestockgenomics.csiro.au/) [44]. The expression level of each miRNA was normalized by the following formula: Normalized expression (NE) = Actual miRNA count/Total count of clean reads * 1,000,000. We removed the miRNAs with a normalized expression level of less than 1 in each of the three libraries and the miRNAs with an estimated probability value of less than 0.95. The fold-change in the expression level and the P-value between two libraries were calculated using the following formulas, respectively:

Fold change = log_2_ (normalized expression 1/ normalized expression 2).

$$\text{p}\left(\text{x|y}\right)={\left(\frac{\text{N}2}{\text{N}1}\right)}^{y}\frac{\left(\text{x}+\text{y}\right)!}{\text{x}!\text{y}!{\left(1+\frac{\text{N}2}{\text{N}1}\right)}^{\left(\text{x}+\text{y}+1\right)}}$$

where the variables x and y represent the normalized miRNA expression levels in the two phases, and N1 and N2 represent the total numbers of clean reads in the two small RNA libraries [45].

### MiRNA validation through stem-loop QPCR

The total RNA of the wool follicles from each sheep was used, and the stem-loop QPCR method [46] was performed for miRNA reverse transcription. The wool follicle samples used in the QPCR analyses were the same as those used in the sequencing experiment. The sheep U6 snRNA was used as an internal control. For each assay, three biological replicates were performed. The 2^-△△Ct^ method was used to analyze the expression level [47]. The reagents include the following: “PrimeScript RT Reagent Kit With gDNA Eraser (Takara)” for reverse transcription and “SYBR Green All-in-One QPCR Mix (Genecopoeia)” for the QPCR assay.

### Prediction of MiRNA target genes and pathways

The target genes of the identified miRNAs were predicted using the RNAhybrid software [48] with the following parameters: helix constraint = 2-7 and energy cut-off = -25. Due to the incomplete information on the sheep reference, the 3’UTR sequence of genes from cattle which are highly evolutionarily homologous with sheep were used [49]. Database: Ensembl Genes 68: *Bos taurus* genes (UMD3.1) (http://asia.ensembl.org). The KEGG pathways were analyzed using the DAVID website [50,51] with the following parameters: Count = 2 and EASE = 1.0. “Count” means the threshold of minimum gene counts belonging to an annotation term, and “EASE” is a modified Fisher Exact P-Value.

## Supporting Information

Figure S1 **Predicted secondary structures of novel miRNAs.**
(PDF)

Table S1 **Identified miRNAs in wool follicles during anagen, catagen, and telogen phases by Solexa sequencing.**
(XLSX)

Table S2 **Sequence information on novel miRNAs in wool follicles.**
(XLSX)

Table S3 **Differentially expressed miRNAs among different wool follicle development phases.**
(DOCX)

Table S4 **Predicted target genes of differentially expressed miRNAs.**
(XLSX)

Table S5 **Predicted KEGG pathways targeted by differentially expressed miRNAs.**
(XLSX)
