# Supplementary material for: Identification of microRNAs in Wool Follicles during Anagen, Catagen, and Telogen Phases in Tibetan Sheep
Source: PLoS One. 2013 Oct 17;8(10):e77801. doi: 10.1371/journal.pone.0077801 (PMC3804049; doi:10.1371/journal.pone.0077801)

oar-novel-1-5P

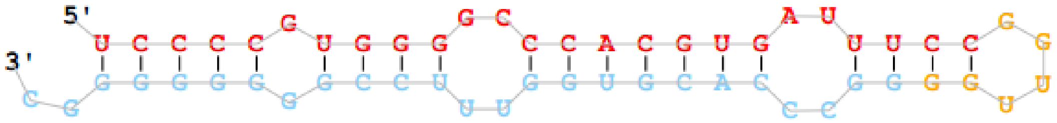

oar-novel-2-3P

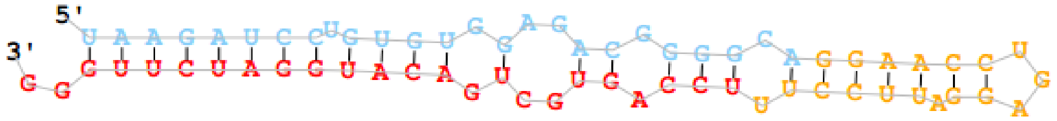

oar-novel-3-3P

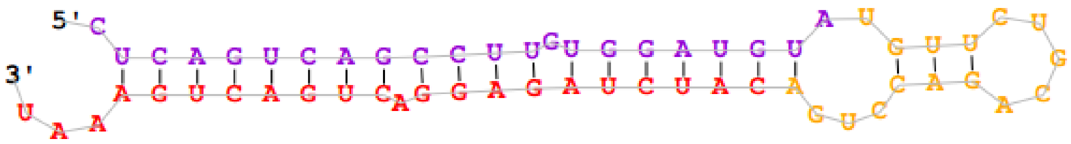

oar-novel-4-3P

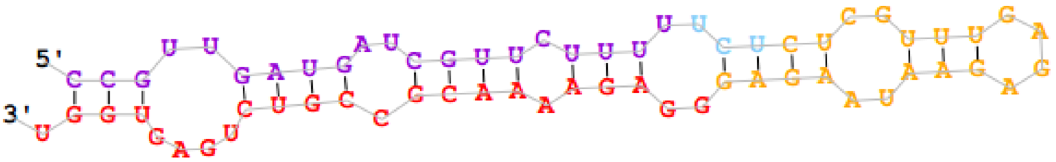

oar-novel-5-5P

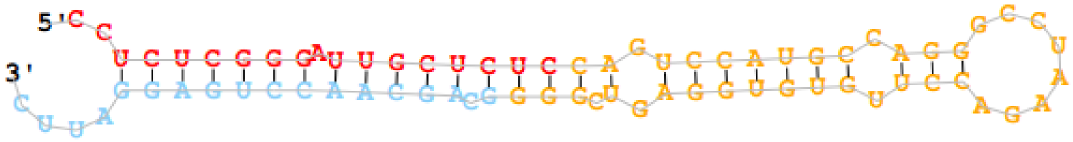

oar-novel-6-3P

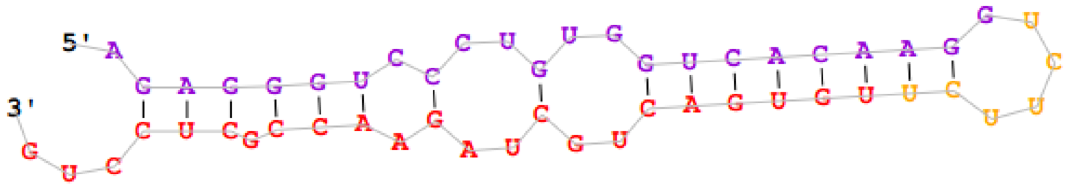

oar-novel-7-3P

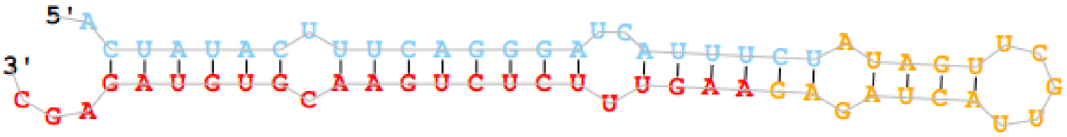

oar-novel-8-5P

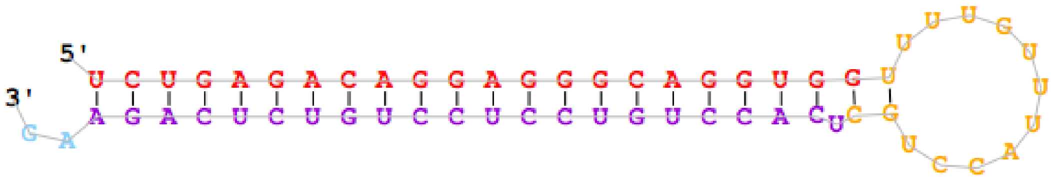

oar-novel-9-3P

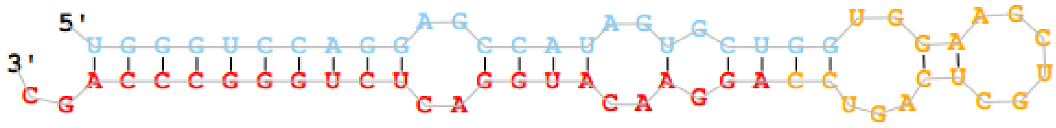

oar-novel-10-5P

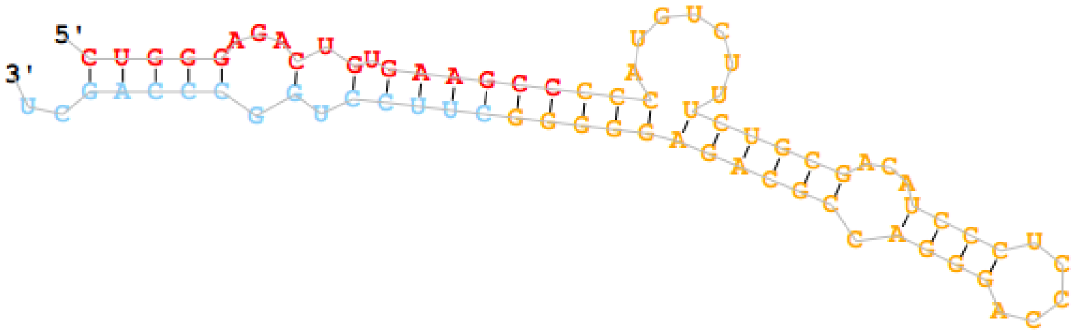

oar-novel-11-3P

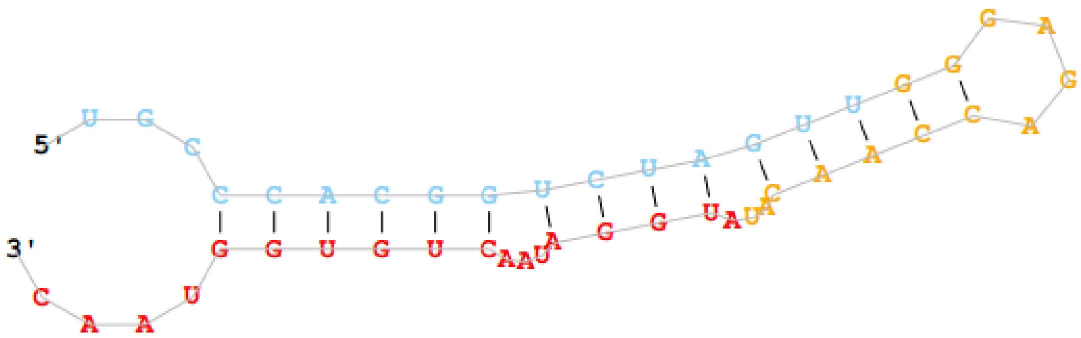

oar-novel-12-3P

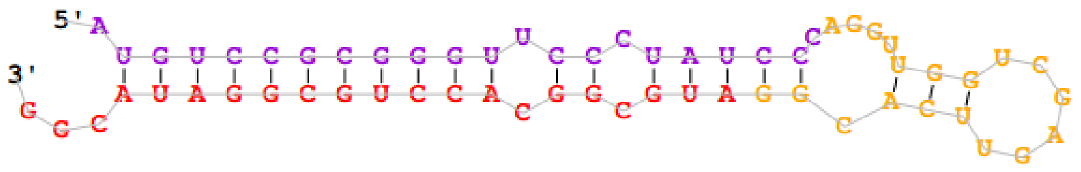

oar-novel-13-5P

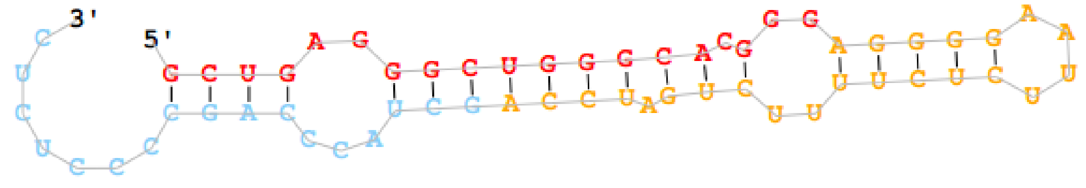

oar-novel-14-3P

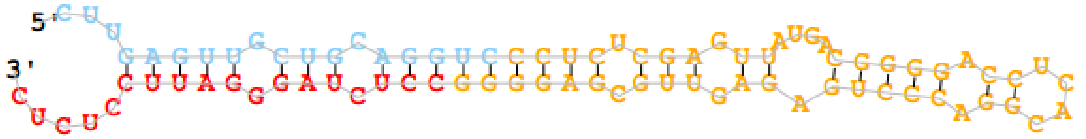

oar-novel-15-3P

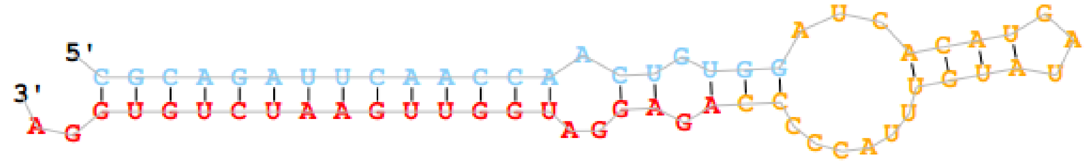

oar-novel-16-3P

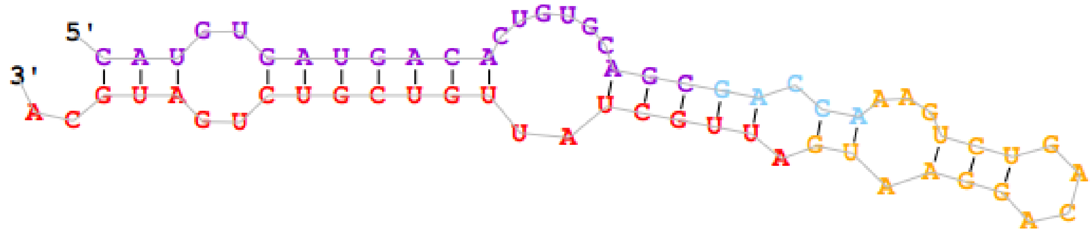

oar-novel-17-3P

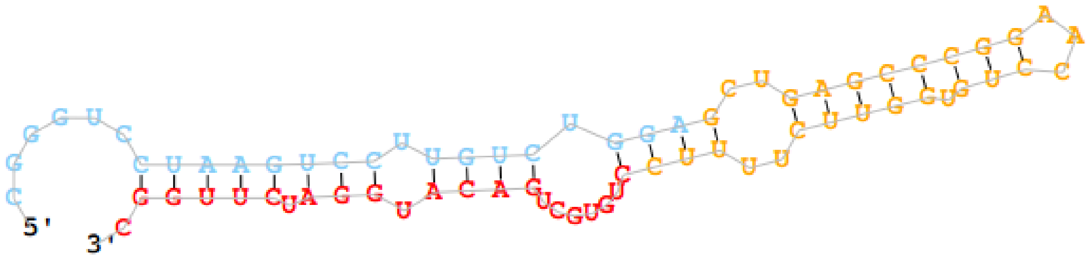

oar-novel-18-3P

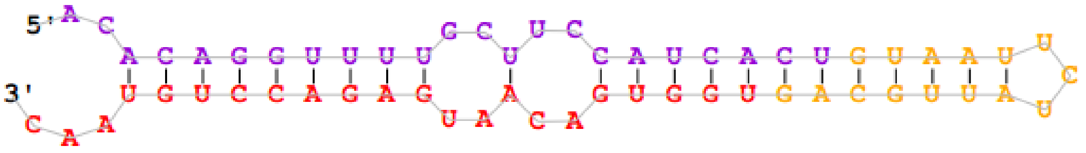

oar-novel-19-3P

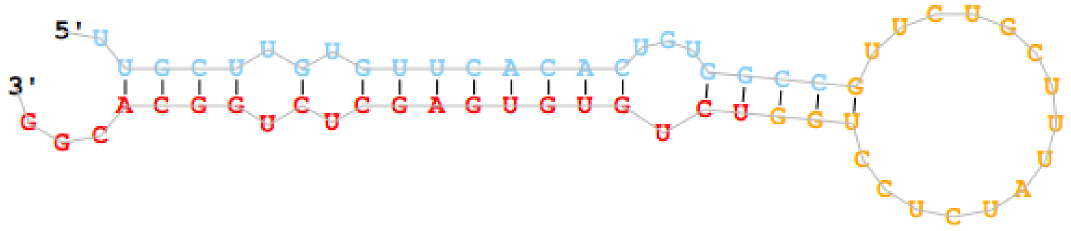

oar-novel-20-3P

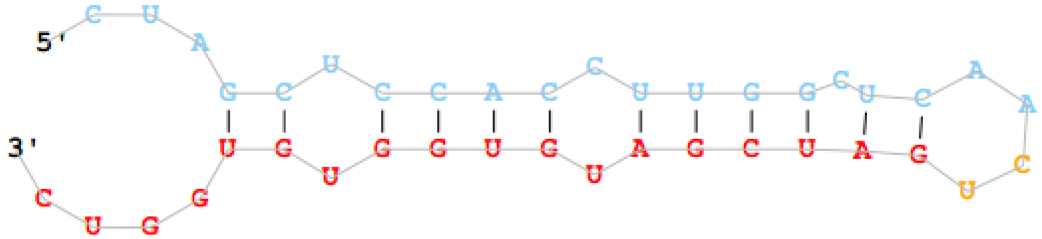

oar-novel-21-5P

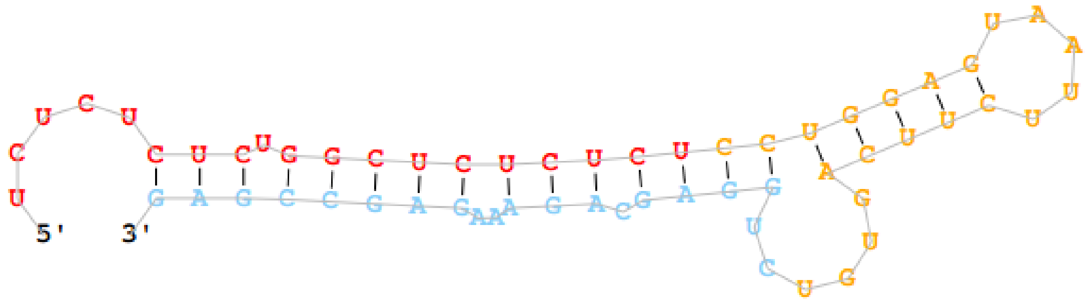

oar-novel-22-3P

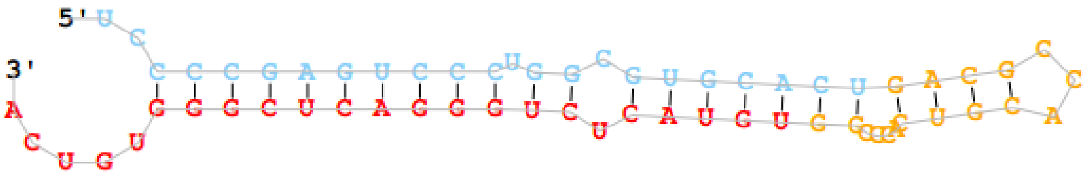

oar-novel-23-3P

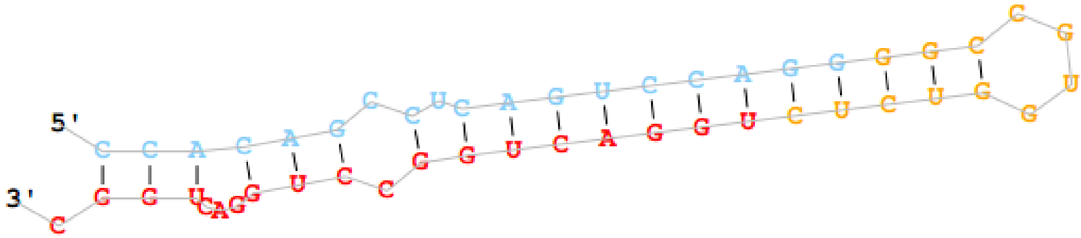

oar-novel-24-3P

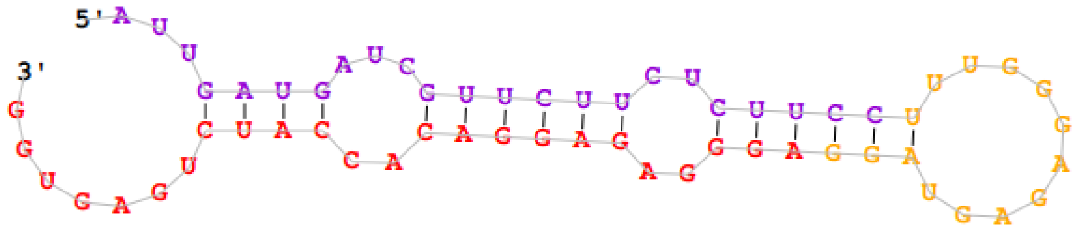

oar-novel-25-3P

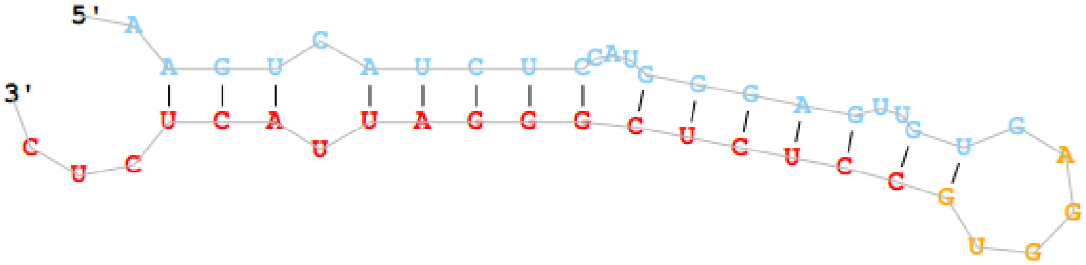

oar-novel-26-3P

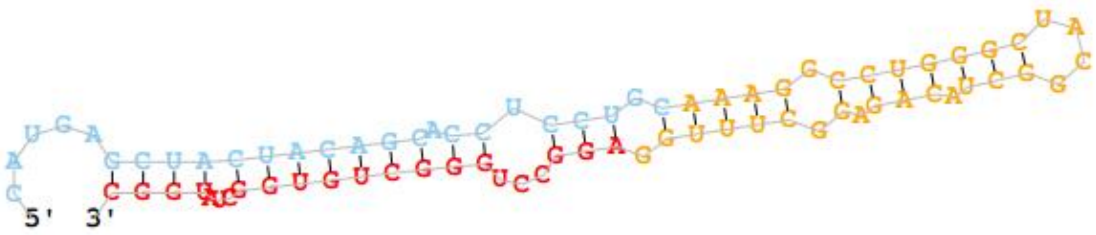

oar-novel-27-5P

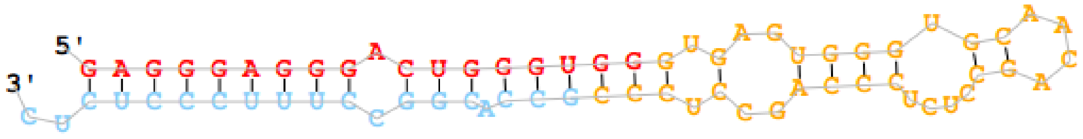

oar-novel-28-3P

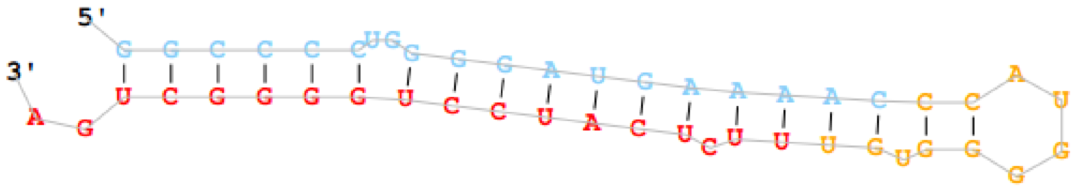

oar-novel-29-5P

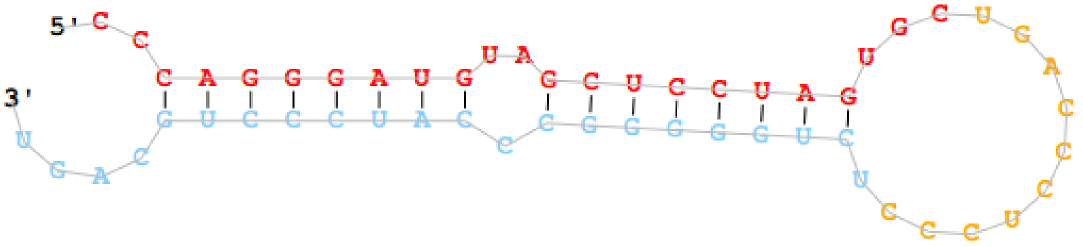

oar-novel-30-5P

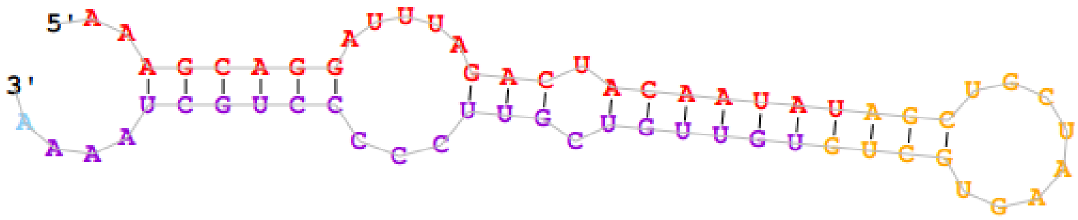

oar-novel-31-5P

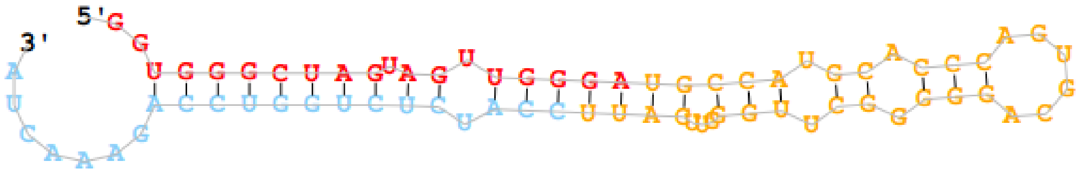

oar-novel-32-3P

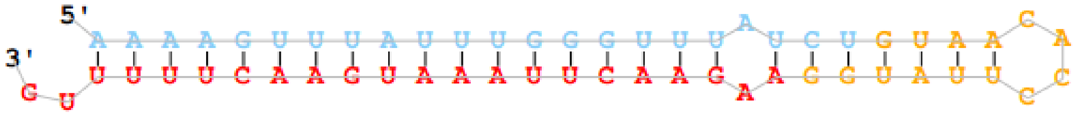

oar-novel-33-3P

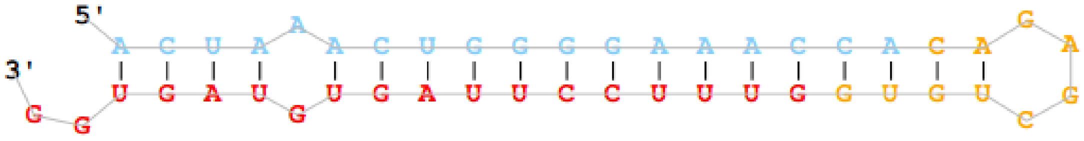

oar-novel-34-3P

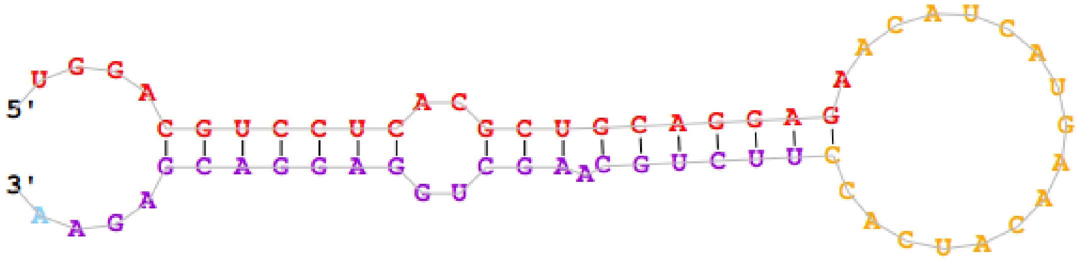

oar-novel-35-3P

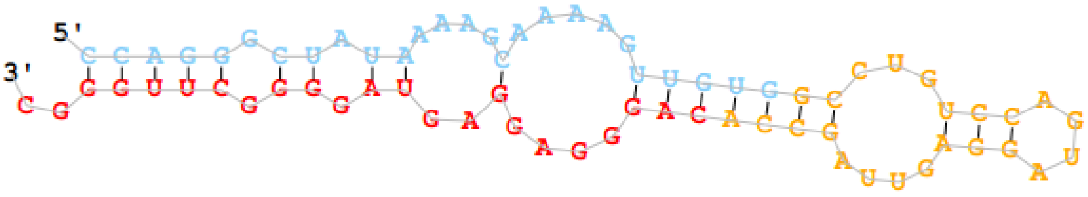

Supplement: Figure S1 — Predicted secondary structures of novel miRNAs. (PDF) [file pone.0077801.s001.pdf]
