# Supplementary material for: Identification of microRNAs in Wool Follicles during Anagen, Catagen, and Telogen Phases in Tibetan Sheep
Source: PLoS One. 2013 Oct 17;8(10):e77801. doi: 10.1371/journal.pone.0077801 (PMC3804049; doi:10.1371/journal.pone.0077801)
Supplement: Table S3 — Differentially expressed miRNAs among different wool follicle development phases. (DOCX) [file pone.0077801.s004.docx]

**Table S3. Differentially expressed miRNAs among different wool follicle development phases**

| **Anagen-Catagen** | | | | |  | **Anagen-Telogen** | | | | |  | **Catagen-Telogen** | | | | |
| --- | --- | --- | --- | --- | --- | --- | --- | --- | --- | --- | --- | --- | --- | --- | --- | --- |
| **miRNA name** | **Normalized Expression level** | | **Change fold** | **Rank** |  | **miRNA name** | **Normalized Expression level** | | **Log2**  **Change fold** | **Rank** |  | **miRNA name** | **Normailzied Expression level** | | **Log2**  **Change fold** | **Rank** |
|  | **Anagen** | **Catagen** |  |  |  |  | **Anagen** | **Telogen** |  |  |  |  | **Catagen** | **Telogen** |  |  |
| oar-let-7a-5P | 66038.41 | 32012.43 | 1.04 | 2 |  | oar-let-7a-5P | 66038.41 | 29733.22 | 1.15 | 2 |  | oar-miR-30e-3P | 445.04 | 3.32 | 7.07 | 46 |
| oar-let-7f-5P | 41098.79 | 13744.00 | 1.58 | 4 |  | oar-let-7f-5P | 41098.79 | 15506.35 | 1.41 | 4 |  | oar-miR-3175-3P | 0.00 | 327.60 | -18.97 | 47 |
| oar-miR-16a-5P | 4407.53 | 2168.61 | 1.02 | 13 |  | oar-miR-103-3P | 6985.91 | 2891.08 | 1.27 | 10 |  | oar-novel-1-5P | 543.61 | 0.00 | 19.64 | 52 |
| oar-let-7g-5P | 4366.05 | 1953.42 | 1.16 | 15 |  | oar-miR-16a-5P | 4407.53 | 1896.28 | 1.22 | 13 |  | oar-novel-2-3P | 38.21 | 414.54 | -3.44 | 55 |
| oar-miR-148a-3P | 2011.73 | 781.33 | 1.36 | 23 |  | oar-let-7g-5P | 4366.05 | 1692.50 | 1.37 | 15 |  | oar-miR-2285b-1-3P | 324.83 | 0.00 | 18.90 | 61 |
| oar-miR-148b-3P | 1483.61 | 470.06 | 1.66 | 28 |  | oar-miR-148a-3P | 2011.73 | 994.86 | 1.02 | 23 |  | oar-miR-2285b-2-3P | 287.26 | 0.00 | 18.72 | 68 |
| oar-miR-320-3P | 1010.17 | 195.54 | 2.37 | 33 |  | oar-miR-148b-3P | 1483.61 | 623.10 | 1.25 | 28 |  | oar-miR-125b-3P | 5.76 | 65.32 | -3.50 | 89 |
| oar-miR-31-5P | 605.43 | 217.05 | 1.48 | 39 |  | oar-let-7i-5P | 1212.17 | 556.65 | 1.12 | 29 |  | oar-novel-5-5P | 5.89 | 79.38 | -3.75 | 98 |
| oar-miR-3175-3P | 443.68 | 0.00 | 19.40 | 47 |  | oar-miR-320-3P | 1010.17 | 192.12 | 2.39 | 33 |  | oar-novel-7-3P | 48.45 | 0.00 | 16.16 | 105 |
| oar-novel-1-5P | 0.00 | 543.61 | -19.34 | 52 |  | oar-miR-143-3P | 647.07 | 267.65 | 1.27 | 34 |  | oar-miR-181a-3P | 3.84 | 38.73 | -3.33 | 106 |
| oar-novel-2-3P | 0.00 | 38.21 | -15.51 | 55 |  | oar-miR-31-5P | 605.43 | 231.84 | 1.38 | 39 |  | oar-miR-33a-5P | 11.71 | 27.72 | -1.24 | 108 |
| oar-miR-542-3P | 187.82 | 92.87 | 1.02 | 57 |  | oar-miR-98-5P | 477.05 | 237.81 | 1.00 | 42 |  | oar-miR-2284b-1-5P | 26.05 | 0.00 | 15.26 | 117 |
| oar-miR-2285b-1-3P | 32.55 | 324.83 | -3.32 | 61 |  | oar-miR-30e-3P | 326.28 | 3.32 | 6.62 | 46 |  | oar-miR-193b-3P | 11.78 | 1.33 | 3.15 | 125 |
| oar-miR-6529-5P | 207.00 | 52.23 | 1.99 | 66 |  | oar-novel-2-3P | 0.00 | 414.54 | -18.95 | 55 |  | oar-miR-122-5P | 0.70 | 20.43 | -4.86 | 132 |
| oar-miR-1-3P | 59.11 | 129.36 | -1.13 | 67 |  | oar-miR-2285b-1-3P | 32.55 | 0.00 | 15.58 | 61 |  | oar-novel-10-5P | 31.24 | 0.00 | 15.52 | 133 |
| oar-miR-2285b-2-3P | 0.00 | 287.26 | -18.42 | 68 |  | oar-miR-6529-5P | 207.00 | 45.36 | 2.19 | 66 |  | oar-miR-331-5P | 0.00 | 11.27 | -14.10 | 137 |
| oar-miR-340-5P | 134.53 | 56.45 | 1.25 | 73 |  | oar-miR-340-5P | 134.53 | 55.17 | 1.29 | 73 |  | oar-miR-194-3P | 10.50 | 0.66 | 3.98 | 147 |
| oar-miR-7-5P | 147.98 | 14.59 | 3.34 | 78 |  | oar-miR-374b-5P | 102.97 | 43.24 | 1.25 | 74 |  | oar-miR-2284q-3P | 0.51 | 14.19 | -4.79 | 150 |
| oar-miR-424-5P | 79.11 | 36.23 | 1.13 | 80 |  | oar-miR-7-5P | 147.98 | 22.41 | 2.72 | 78 |  | oar-miR-331-3P | 0.00 | 8.22 | -13.65 | 151 |
| oar-miR-125b-3P | 62.80 | 5.76 | 3.45 | 89 |  | oar-miR-221-3P | 72.23 | 26.33 | 1.46 | 95 |  | oar-miR-2284b-2-5P | 0.38 | 14.32 | -5.22 | 154 |
| oar-miR-221-3P | 72.23 | 17.22 | 2.07 | 95 |  | oar-novel-5-5P | 2.05 | 79.38 | -5.28 | 98 |  |  |  |  |  |  |
| oar-miR-96-5P | 39.60 | 16.00 | 1.31 | 103 |  | oar-miR-503-5P | 46.73 | 13.40 | 1.80 | 104 |  |  |  |  |  |  |
| oar-miR-503-5P | 46.73 | 15.04 | 1.64 | 104 |  | oar-novel-7-3P | 26.32 | 0.00 | 15.28 | 105 |  |  |  |  |  |  |
| oar-miR-181a-3P | 29.60 | 3.84 | 2.95 | 106 |  | oar-miR-17-3P | 0.82 | 38.86 | -5.56 | 112 |  |  |  |  |  |  |
| oar-miR-33a-5P | 25.99 | 11.71 | 1.15 | 108 |  | oar-miR-2284b-1-5P | 29.76 | 0.00 | 15.45 | 117 |  |  |  |  |  |  |
| oar-miR-17-3P | 0.82 | 22.53 | -4.78 | 112 |  | oar-novel-8-5P | 11.23 | 26.86 | -1.26 | 118 |  |  |  |  |  |  |
| oar-miR-2284n-3P | 42.55 | 0.00 | 16.02 | 122 |  | oar-miR-2284n-3P | 42.55 | 0.00 | 15.97 | 122 |  |  |  |  |  |  |
| oar-miR-193b-3P | 26.81 | 11.78 | 1.19 | 125 |  | oar-miR-193b-3P | 26.81 | 1.33 | 4.34 | 125 |  |  |  |  |  |  |
| oar-miR-2285b-5-3P | 32.30 | 0.00 | 15.62 | 131 |  | oar-miR-2285b-5-3P | 32.30 | 0.00 | 15.57 | 131 |  |  |  |  |  |  |
| oar-miR-122-5P | 10.17 | 0.70 | 3.85 | 132 |  | oar-miR-330-3P | 20.58 | 3.78 | 2.44 | 134 |  |  |  |  |  |  |
| oar-novel-10-5P | 0.00 | 31.24 | -15.22 | 133 |  | oar-novel-11-3P | 0.00 | 7.29 | -13.12 | 138 |  |  |  |  |  |  |
| oar-miR-330-3P | 20.58 | 4.80 | 2.10 | 134 |  | oar-miR-3604-3P | 13.53 | 2.72 | 2.31 | 141 |  |  |  |  |  |  |
| oar-miR-331-5P | 15.25 | 0.00 | 14.54 | 137 |  | oar-miR-2284q-3P | 1.15 | 14.19 | -3.63 | 150 |  |  |  |  |  |  |
| oar-novel-11-3P | 0.00 | 16.83 | -14.33 | 138 |  | oar-miR-2284b-2-5P | 0.00 | 14.32 | -14.09 | 154 |  |  |  |  |  |  |
| oar-miR-877-5P | 10.82 | 4.03 | 1.42 | 139 |  |  |  |  |  |  |  |  |  |  |  |  |
| oar-miR-3604-3P | 13.53 | 6.27 | 1.11 | 141 |  |  |  |  |  |  |  |  |  |  |  |  |
| oar-miR-331-3P | 7.62 | 0.00 | 13.54 | 151 |  |  |  |  |  |  |  |  |  |  |  |  |
| oar-miR-1277-5P | 5.66 | 0.00 | 13.11 | 166 |  |  |  |  |  |  |  |  |  |  |  |  |
| oar-miR-365-3P | 5.16 | 0.00 | 12.98 | 191 |  |  |  |  |  |  |  |  |  |  |  |  |

Note: All the miRNAs meet the criteria P ≤ 0.01.
